# Statistics of Operations for Cancer

**Published:** 1853-09

**Authors:** 


*Statistics of Operations for Cancer.* By Professor Paget. In a recent note to The Lancet, Mr. Paget qualifies a remark which he had made in his lectures at the College of Surgeons, "that persons operated upon for cancer die upon an average thirteen months sooner than those upon whom no operation has been performed," by stating that he referred only to scirrhous cancer of the breast. He says —

In such cases, I believe that the general average duration of life, after the patient's first observation of the disease, is forty-nine months; that the average life of those whose breasts are removed, and who survive the effects of the operation, is forty-three months; and that the average life of those in whom the disease is allowed to run its course is about fifty-five months.

In the second lecture, I said that the general result of operations for medullary cancers is very different; and that, although they are so seldom long survived, that they are generally considered to be less beneficial than the operations for scirrhous cancers of the breast, yet on the whole they are more so. The general average of life of persons affected with medullary cancer of the eye, testicle, breast, bones, or other external organ, may be reckoned at about twenty-four months from their first notice of the disease; but I believe the average for those from whom the primary disease is removed, and who do not die in consequence of the operation, is about thirty-four months; while the average for those in whom the disease is allowed to run its course is scarcely more than a year.

In the third lecture I expressed the belief that, on the whole, the operation for epithelial cancers is even more effective in prolonging life than the operation for medullary cancers; but that the wide diversities in the duration of life amongst those affected with this form of cancer makes it very difficult, at present, to deduce such an average as may be relied on. And I would repeat what I said in one lecture respecting all these averages—namely, that such general results serve only general considerations in the treatment of particular cases of cancer. They may justly determine a general rule of action, but it can be only such a rule as must admit of numerous exceptions. In many cases of scirrhous cancer there are sufficient reasons for operating; and, in many cases of medullary and epithelial cancers, reasons as sufficient for refraining. The right course must, in each case, be determined by a just appreciation of all the conditions each presents.—*The Lancet.*

